# Facile Fabrication of Size-Tunable Core/Shell Ferroelectric/Polymeric Nanoparticles with Tailorable Dielectric Properties via Organocatalyzed Atom Transfer Radical Polymerization Driven by Visible Light

**DOI:** 10.1038/s41598-018-38039-8

**Published:** 2019-02-12

**Authors:** Ning You, Chenxi Zhang, Yachao Liang, Qi Zhang, Peng Fu, Minying Liu, Qingxiang Zhao, Zhe Cui, Xinchang Pang

**Affiliations:** 1 School of Materials Science and Engineering, Zhengzhou University, Zhengzhou, 450001 China; 2 Engineering Laboratory of High Performance Nylon Engineering Plastics of CPCIF, Zhengzhou University, Zhengzhou, 450001 China; 3 Henan Joint International Research Laboratory of Living Polymerizations and Functional Nanomaterials, Zhengzhou University, Zhengzhou, 450001 China

## Abstract

An unconventional but facile approach to prepare size-tunable core/shell ferroelectric/polymeric nanoparticles with uniform distribution is achieved by metal-free atom transfer radical polymerization (ATRP) driven by visible light under ambient temperature based on novel hyperbranched aromatic polyamides (HBPA) as a functional matrix. Cubic BaTiO_3_/HBPA nanocomposites can be prepared by *in-situ* polycondensation process with precursors (barium hydroxide (Ba(OH)_2_) and titanium(IV) tetraisopropoxide (TTIP)) of ferroelectric BaTiO_3_ nanocrystals, because precursors can be selectively loaded into the domain containing the benzimidazole rings. At 1200 °C, the aromatic polyamide coating of cubic BaTiO_3_ nanoparticles are carbonized to form carbon layer in the inert environment, which prevents regular nanoparticles from gathering. In addition, cubic BaTiO_3_ nanoparticles are simultaneously transformed into tetragonal BaTiO_3_ nanocrystals after high temperature calcination (1200 °C). The outer carbon shell of tetragonal BaTiO_3_ nanoparticles is removed via 500 °C calcination in air. Bi-functional ligand can modify the surface of tetragonal BaTiO_3_ nanoparticles. PMMA polymeric chains are growing from the initiating sites of ferroelectric BaTiO_3_ nanocrystal surface via the metal-free ATRP technique to obtain core/shell ferroelectric BaTiO_3_/PMMA hybrid nanoparticles. Changing the molar ratio between benzimidazole ring units and precursors can tune the size of ferroelectric BaTiO_3_ nanoparticles in the process of polycondensation, and the thickness of polymeric shell can be tailored by changing the white LED irradiation time in the organocatalyzed ATRP process. The dielectric properties of core/shell BaTiO_3_/PMMA hybrid nanoparticles can be also tuned by adjusting the dimension of BaTiO_3_ core and the molecular weight of PMMA shell.

## Introduction

Core/shell nanostructures consisting of an inorganic nanoparticle core and inorganic or organic shell have been broadly studied, not only as a strategy for improving the stability and changing the surface chemistry of inorganic core nanoparticles, but also as a method for obtaining unique chemical and physical properties, which is impossible from individual materials alone^1,2^. For example, when the surface of Au nanoparticles is coated by organic ligands comprising thiol groups as shell, unique optical properties can be generated^3,4^. Besides small molecule ligands, functional polymeric coatings as shell on core nanoparticle surfaces are also widely used to prepare interfaces with special properties and characteristics to make them to interact with specific environment^5,6^. For example, the coating shell can be utilized to reduce undesirable interactions or enhance desired interactions^7^. Different from inorganic shell growing from the surface of nanoparticles, various different approaches have been utilized for the preparation of functional polymer shells, such as *in situ* polymerization from the functional surface of nanoparticles^8^, direct attaching functional polymer ligands onto surfaces of core nanoparticles^2,9^, layer-by-layer functional polymer deposition^10,11^, and *in situ* fabrication of inorganic nanoparticles using functional polymeric chains as ligands. However, in all of these strategies, it is challenging to control the growth of functional polymeric shell^12^.

Owing to its room temperature ferroelectric behaviors and high permittivity^13^, BaTiO_3_ nanomaterials are one of the most widely investigated ferroelectric materials^14,15^. BaTiO_3_ nanomaterials can be used in various fields, such as multilayer capacitors, electro-optical devices, actuators and so on^16^. The ferroelectric behaviors and dielectric properties of BaTiO_3_ materials depend heavily on morphologies, structures, nanoparticle sizes, crystalline structures, surface chemistry and so on^16,17^. In many cases, nanomaterials with low dielectric loss, high energy storage capability and high permittivity are highly desirable, especially for applications in modern electric and electronics fields^17^. Core/shell BaTiO_3_/polymer nanoparticles combine the characteristics of BaTiO_3_ materials and polymeric shell, showing the high permittivity, low dielectric loss and easily processing^18^. However, in conventional routes, such as melt blending or solution mixing, a lot of issues can be raised such as inhomogeneity and aggregation resulting in undesirable properties^18^. A facile approach to fabricate size-tunable core/shell ferroelectric/polymeric nanoparticles with uniform distribution is of great interest for a variety of application areas mentioned above.

Hyperbranched polymers (HBPA) possessing a three-dimensional molecular architecture with a highly branched backbone and many terminal functional groups exhibit different characteristics from the linear polymer analogues, such as high solubility and low solution viscosity^19^. In addition, aromatic polyamides have been broadly utilized as frequently used high-performance polymeric materials in the electronics and aerospace fields owing to their excellent properties, such as excellent mechanical properties, high thermal stability, low relative permittivity, low dielectric constant, low thermal expansion, high breakdown voltage, long-term stability, good hydrolytic stability and so on^20^. Unfortunately, most linear aromatic polyamides are generally characterized by poor processability^19^. It is worthwhile to bring hyperbranched structures into aromatic polyamides to improve the poor fabricability caused by the rigid repeating unit in the linear aromatic polyamides. Furthermore, the benzimidazole rings are widely utilized in the preparation HBPA because of its stability, asymmetric structure, stiffness and complexing abilities of metal ions^19^. Due to its unique chemical structures and synthesis conditions, HBPA containing benzimidazole rings are the outstanding choice as a functional polyamide matrix and the following carbon coating as protecting layer for the preparation of tetragonal BaTiO_3_ nanocrystals^21^.

Over the past several decades, due to ability of precision design and synthesis of a variety of polymers, atom transfer radical polymerization (ATRP) techniques have been one of the most effective approaches^22^. In the traditional ATRP techniques, transition-metal catalysts were used to mediate redox equilibrium process^23,24^, introducing the catalyst purification challenges and contamination and impeding their wide applications in biomaterials, microelectronics, functional inorganic/organic core/shell nanocomposites, etc^25^. Despite a lot of investigations in reducing catalyst loading and facilitating the metal catalyst removal^25^, it is still a challenge to extensively use conventional ATRP for the preparation of core/shell inorganic/polymer hybrid nanoparticles. Metal-free ATRP technique remains highly desirable to circumvent the removal of metal catalyst, avoid contamination and reduce toxicity concerns^26–28^.

Here we report an unconventional but facile approach to fabricate size-tunable core/shell ferroelectric/polymeric nanoparticles with uniform distribution via metal-free ATRP driven by visible light under ambient temperature based on novel HBPA as a functional matrix. Ba(OH)_2_ and TTIP were used as precursors of ferroelectric BaTiO_3_ nanocrystals that were selectively loaded into the domain containing benzimidazole rings by coordination interactions between benzimidazole rings and precursors^19,29^, then equimolar trimesic acid and 2-(4-aminophenyl)-1H-benzimidazol-5-amine as monomers were subjected to *in-situ* polycondensation with precursors to form BaTiO_3_ nanoparticles, embedded in the HBPA matrix. At 1200 °C under the inert environment, the aromatic polyamide as coating of cubic BaTiO_3_ nanoparticles were carbonized to form carbon layer, which acted as protecting shell to prevent nanoparticles from gathering. In addition, cubic BaTiO_3_ nanoparticles were simultaneously transformed into tetragonal BaTiO_3_ after 1200 °C calcination. Then the carbon layer on the surface of tetragonal BaTiO_3_ nanoparticles was removed via calcination under relative low temperature (500 °C in air). In addition, the bi-functional ligands used as the metal-free ATRP initiator were synthesized via modifying the hydroxyl group of 12-hydroxydodecanoic acid by 2-bromophenylacetyl bromide. Then the bi-functional ligands were used for the surface modification of tetragonal BaTiO_3_ nanocrystals. PMMA polymeric chains were growing from the initiating sites of ferroelectric BaTiO_3_ nanocrystal surface by initiating the polymerization of methyl methacrylate (MMA) monomers via the metal-free ATRP technique to obtain core/shell ferroelectric BaTiO_3_/PMMA hybrid nanoparticles, composed of ferroelectric BaTiO_3_ nanocrystals as core and PMMA polymeric chains as shell with different dimensions, 5,10-di(1-naphthyl)-5,10-dihydrophenazine as an organic photocatalyst under a white LED irradiation at ambient temperature. The dimensions of ferroelectric BaTiO_3_ nanoparticles can be adjusted via changing the molar ratio between benzimidazole ring units and precursors in the polycondensation process, and the thickness of polymeric shell can be also tailored by changing the white LED irradiation time within the ATRP process. The dielectric properties of core/shell BaTiO_3_/PMMA hybrid nanoparticles are tunable by adjusting the dimension of BaTiO_3_ core and the molecular weight of PMMA shell.

## Experimental Section

### Materials

Trimesic acid (TMA) and 2-(4-aminophenyl)-1H-benzimidazol-5-amine (APBIA) were purchased from Aladdin Co. (China) and used as starting materials without further purification. THF and NMP (tetrahydrofuran, N-methyl-2-pyrrolidone) were procured from Guoyao Co. (China), distilled under calcium hydride and reduced pressure before use. Barium hydroxide (Ba(OH)_2_, ≥ 95.0%) and titanium(IV) isopropoxide (TTIP precursor, ≥ 97.0%) without further purification were purchased from Sigma-Aldrich. The white LED strips (4 W) were purchased from LEDlightinghut.com. All reactions were vigorously stirred in front of white LED while cooling with compressed air to maintain ambient temperature (the light intensity: 0.61 μW/cm^2^).

### Characterizations

^1^H-NMR and ^13^C-NMR characterizations were carried out with a Bruker DPX-400 (400 MHz), deuterated dimethyl sulfoxide (DMSO-*d6*) and trifluoroacetic acid (TFA-*d*) as solvents, and tetramethyl-silane (TMS) used as an internal reference. The FT-IR measurements were conducted on a NICOLET 460 spectrometer (KBr pellet) (the range: 4000-400 cm^−1^; the resolution: 4 cm^−1^). The concentration of polyamides in KBr pellet (200 mg) was about 1%. Shapes and dimensions of cubic BaTiO_3_/HBPA nanocomposites, carbon-capped tetragonal BaTiO_3_ nanoparticles, tetragonal BaTiO_3_ nanoparticles after removal of carbon shell, tetragonal BaTiO_3_ nanoparticles coated with the metal-free ATRP initiators and core/shell ferroelectric BaTiO_3_/PMMA hybrid nanoparticles, were observed via transmission electron microscope (TEM) (JEOL 1200EX; characterized at 80 kV). The preparation process for TEM characterization samples as follow: (1) cubic BaTiO_3_/HBPA nanocomposites were prepared into the bulk under hot-pressing, following by the preparation of TEM samples using microtome (ULTRACUT E, Reichert-Jung) (thickness: < 100 nm); (2) TEM characterization samples of carbon-capped tetragonal BaTiO_3_ nanoparticles, tetragonal BaTiO_3_ nanoparticles after removal of carbon shell, were prepared fabricated by drop-casting method (a drop of nanoparticle ethanol solution, on TEM grids); (3) TEM characterization samples of tetragonal BaTiO_3_ nanoparticles capped with the metal-free ATRP initiators were fabricated via applying nanocrystals toluene solution (volume: ~10 μL; c: 1 mg/mL) on regular TEM grids; (4) TEM characterizaion samples of core/shell ferroelectric BaTiO_3_/PMMA hybrid nanoparticles were prepared by using a drop of nanoparticle toluene (volume: ~10 μL; c: 1 mg/mL) on regular TEM grids, and then dried under room temperature. In addition, for the TEM characterization of polymeric shell on the surface of BaTiO_3_ nanoparticles, the PMMA shell were stained via RuO_4_ (ruthenium tetraoxide). X-ray diffraction (XRD) characterization was used to confirm the crystal structures of samples (SCINTAG XDS-2000; Cu Kα radiation). In addition, the morphology of nanocomposites and the energy dispersive spectroscopy (EDS) characterization of samples were carried out via field emission scanning electron microscopy (FE-SEM; FEI Quanta 250). Molecular weight (MW) and polydispersity index (PDI) of PMMA grafting chains were characterized by GPC (Agilent1100 with a G1310A pump, a G1314A variable wavelength detector and a G1362A refractive detector). THF was used as eluent at 35 °C (1.0 mL/min). All the columns composed of two 5 μm LP gel mixed bed columns (molecular range: 200-3 × 10^6^ g/mol) and one 5 μm LP gel column (500 Å, molecular range: 500-2 × 10^4^ g/mol) were calibrated by PS standard samples. The weight fractions of the organic shell in tetragonal BaTiO_3_ nanocrystals coated with the metal-free ATRP initiators and core/shell ferroelectric BaTiO_3_/PMMA hybrid nanoparticles were measured via TGA (thermogravimetric analysis characterization; TA Instrument TGA Q 50). In order to measure the dielectric properties of core/shell tetragonal BaTiO_3_/PMMA hybrid nanoparticles and corresponding PMMA shell in the microwave frequency range, all samples were compressed into toroidal shape (inner diameter: 3.00 mm; outer diameter: 7.00 mm). The complex permittivity of samples was characterized by Vector Network Analyzer (Anritsu 37347 C) incorporating a *S*-parameter test set. The *S*-parameters were characterized by utilizing the coaxial transmission**/**reflection method, and converted to complex permittivity via Nicholson-Ross-Weir algorithm^30^.

### Preparation of cubic BaTiO_3_/HBPA nanocomposites

**Figure 1:**
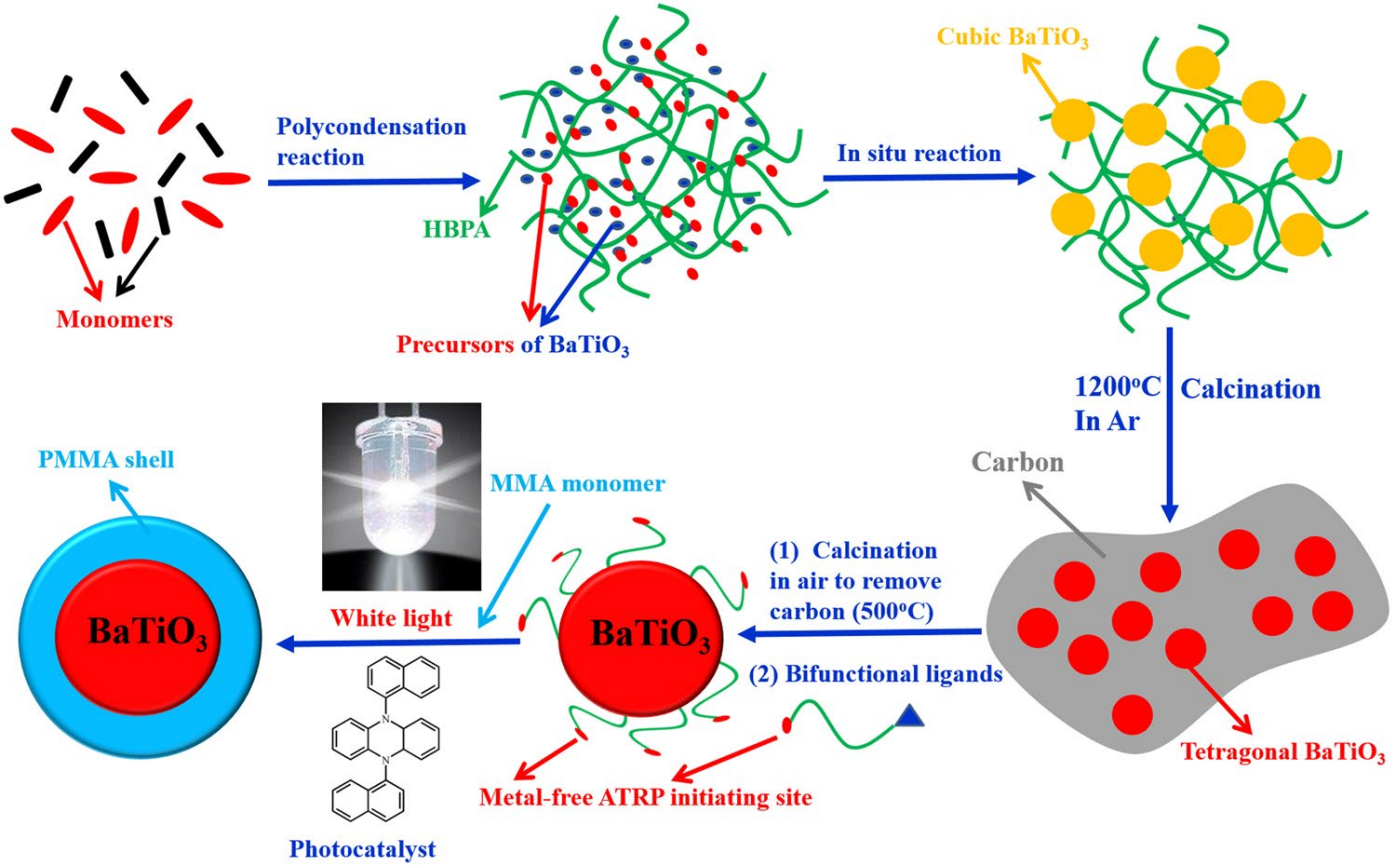
Scheme for the preparation process of core/shell ferroelectric BaTiO_3_/PMMA hybrid nanoparticles by metal-free ATRP driven by visible light based on novel hyperbranched aromatic polyamides (HBPA) as functional matrix.

As shown in Figs 1 and S1, cubic BaTiO_3_/hyperbranched polyamide (HBPA) with benzimidazole rings were synthesized through a one-step procedure^14^ with equimolar monomers of trimesic acid and 2-(4-aminophenyl)-1H-benzimidazol-5-amine. The NMP solution of TMA (10 mmol) and APBIA (10 mmol) was added in a dry 250 mL flask (three-necked, round bottom) with a magnetic stirrer and a condenser pipe. The triphenyl phosphite and pyridine as the condensing agents were added into the flask. The reaction mixture was then heated to reflux under the nitrogen atmosphere in an oil bath of 90 °C. After 3 h, Ba(OH)_2_ (10 mmol) and TTIP (10 mmol) were added into the reaction solution (the molar ratio of precursors to benzimidazole ring = 1:1). After another 1 h reaction, pale yellow precipitates of cubic BaTiO_3_/HBPA were formed by slowly pouring the reaction solution into methanol under uniformly stirring. The precipitates were purified by washing successively with methanol and water, and then dried to constant weight in a vacuum oven at 80 °C.

### Synthesis of carbon-capped tetragonal BaTiO_3_ nanoparticles

After 2 h calcination at 1200 °C under argon atmosphere, the cubic structures of BaTiO_3_ nanoparticles embedded into HBPA matrix were transferred to tetragonal BaTiO_3_ nanoparticles, while the outer coating of HBPA was transferred into carbon layers as protecting shell of the BaTiO_3_ nanoparticles to prevent the nanocrystals from forming larger irregular structures during the calcination process.

### Preparation of carbon-free coated tetragonal BaTiO_3_ nanoparticles

The carbon layer of core/shell structure tetragonal BaTiO_3_/carbon nanoparticles prepared in the previous step was removed by 500 °C calcination (5 h) under air. Since the tetragonal BaTiO_3_ nanoparticles had a thermodynamically stable crystalline structure, their shapes were kept after 500 °C calcination. The color of the nanoparticles was transferred from black to gray as the carbon layer were removed.

### Synthesis of 5,10-di(1-naphthyl)-5,10-dihydrophenazine as photocatalyst

5,10-di(1-naphthyl)-5,10-dihydrophenazine as a photocatalyst was^27^ synthesized according to reported literature^27^. In a dried vacuum flask, 5,10-dihydrophenazine (911 mg, 5.00 mmol), NaOtBu (1.92 g, 20.0 mmol), RuPhos (46.7 mg, 0.10 mmol), RuPhos precatalyst (81.7 mg, 0.10 mmol) and 1-bromonaphthalene (4.14 g, 20.0 mmol) were added in 10.0 mL anhydrous dioxane. This flask was sealed under argon and heated at 110 °C for 48 h. When reaction mixture was cooled to room temperature, 300 mL CH_2_Cl_2_ and 300 mL H_2_O were added to the reaction flask to produce precipitate. The as-prepared crude product was purified via washing with CH_2_Cl_2_, and the yellow powder as final product was obtained (0.075 g). ^1^H-NMR (C_6_D_6_, 400 MHz) δ 8.64–8.54 (m, 2 H), 7.73–7.63 (m, 4 H), 7.47 (m, 2 H), 7.33–7.22 (m, 6 H), 6.12–6.03 (dd, 4 H), 5.70–5.63 (dd, 4 H).

### Synthesis of metal-free ATRP initiators with bi-functional groups

The metal-free ATRP initiators with bi-functional groups^7^ were synthesized by modifying the hydroxyl group in 12-hydroxydodecanoic acid by 2-bromophenylacetyl bromide (Fig. S5). The typical process is as follows: anhydrous 12-hydroxydodecanoic acid (12 mmol) was dissolved in anhydrous 1-methyl-2-pyrrolidione (NMP, 120 mL), and then cooled to 0 °C. 2-Bromophenylacetyl bromide (80 mL) was added dropwise into the reaction solution with magnetic stirring. After that, the reaction temperature was maintained at 0 °C for 2 h, and then slowly increased to room temperature. The reaction solution was maintained at ambient temperature to react for another 24 h. The as-prepared brown solution was concentrated by vacuum distillation. The resulting crude product was diluted with 200 mL dichloromethane, followed by washing with DI water (3 × 100 mL). The organic layer was concentrated by vacuum distillation to obtain the final bi-functional metal-free ATRP initiators (10.1 g, yield = 72.3%). ^1^H-NMR: δ = 6.33–7.31 ppm (the protons on phenyl ring), δ = 5.55 ppm (Br-C***H***(phenyl)-), δ = 4.09 ppm (-O-C***H***_2_-CH_2_-), and δ = 1.29–2.23 ppm (the protons on methene). ^13^C-NMR: δ = 127.2–134.6 ppm (the carbon atoms on phenyl ring), δ = 52.3 ppm (Br-***C***H(phenyl)-), δ = 173.2 ppm (Br-CH(phenyl)-***C***OO-), δ = 64.8 ppm (-O-***C***H_2_-CH_2_-), δ = 177.6 ppm (-CH_2_-CH_2_-***C***OOH) and δ = 24.5–36.2 ppm (the carbon atoms on methene). The bi-functional ligands were characterized by FT-IR: 1735 cm^−1^ (ν_C=O_), 2933 cm^−1^ (ν_C-H_), 1156 cm^−1^ (ν_C-O-C_), 1037 and 1108 cm^−1^ (coupled ν_C-C_ and ν_C-O_), 2500–3500 cm^−1^ (ν_O-H_).

### Fabrication of tetragonal BaTiO_3_ nanoparticles coating with metal-free ATRP initiators

Preparation of tetragonal BaTiO_3_ nanoparticles coating with metal-free ATRP initiators was carried out by functional ligand absorption. In a typical process, tetragonal BaTiO_3_ nanoparticles without carbon coating (100 mg) were firstly dispersed by ultrasonic instrument into toluene (100 mL) for 2 h. Subsequently, bifunctional metal-free ATRP initiator (50 mg) were dissolved into the solution system by other 2 h ultrasonic dispersion to obtain tetragonal BaTiO_3_ nanoparticles capped with metal-free ATRP initiating sites. Finally, the centrifuging process was used for the removal of excess ligands (10000 rpm, 20 min). Owing to bi-functional ligands as shell layer, the system of tetragonal BaTiO_3_ nanoparticles/ligands can be dissolved into organic solvent (e.g., toluene) to form uniform solution. In order to investigate stability of bi-functional ligands on the surface of BaTiO_3_ nanoparticles in organic solvent (e.g., toluene), 20 mL of toluene solution of BaTiO_3_ nanoparticles coated with bi-functional ligands was prepared (1 mg/mL). After the solution was vigorously stirred for 48 h at room temperature, BaTiO_3_ nanoparticles coated with bi-functional ligands was obtained by centrifuging (10000 rpm, 20 min), and residual solution was characterized by ^1^H-NMR after toluene solvent was completely removed under vacuum. At the same time, BaTiO_3_ nanoparticles coated with bi-functional ligands were characterized by TGA again to confirm weight fraction of the bi-functional ligands.

### Fabrication of core/shell tetragonal BaTiO_3_/PMMA hybrid nanoparticles by metal-free ATRP driven by visible light

Core/shell tetragonal BaTiO_3_/PMMA hybrid nanoparticles were fabricated by metal-free ATRP driven by white LED light^31^. PMMA polymeric chains were growing from the initiating sites on the surface of tetragonal BaTiO_3_ by the living polymerization of MMA monomers, 5,10-di(1-naphthyl)-5,10-dihydrophenazine was used as the photocatalyst under white LED light irradiation at room temperature. In a typical procedure, an ampule charged with a small stir bar, MMA (8 mL), visible light photocatalyst (0.1 mol%), BaTiO_3_ nanoparticle-based initiators (50 mg), and 8 mL *N*, *N*-dimethylacetamide (DMA) was de-gassed via three freeze-thaw cycles in liquid nitrogen, then sealed at ambient temperature. The reaction was vigorously stirred in front of white LED while cooling with compressed air to maintain ambient temperature. The ampule was taken out from the white LED irradiation at different desired time to stop the polymerization. The mixture solution was then diluted by acetone, and then precipitated in the mixed solvent (methanol/water, v/v = 1/1). After centrifugation, the final product was purified via dissolution/precipitation twice by acetone and methanol/water, and then dried at 50 °C in vacuum for 12 h.

### Control experiments by metal-free ATRP driven by visible light

In order to compare with initiators on the surface of tetragonal BaTiO_3_ nanoparticles, free bi-functional initiators were added in the polymerization reaction system to initiate MMA monomers and grow free PMMA polymeric chains via the metal-free ATRP process, 5,10-di(1-naphthyl)-5,10-dihydrophenazine used as the photocatalyst under white LED light irradiation at room temperature. In a typical procedure, an ampule charged with MMA (8 mL), 5,10-di(1-naphthyl)-5,10-dihydrophenazine (0.1 mol%), BaTiO_3_ nanoparticle-based initiators (50 mg), free bi-functional ligands (10 mg), and 8 mL *N*, *N*-dimethylacetamide (DMA) was degassed via three freeze-thaw cycles in liquid nitrogen, then sealed at ambient temperature. Then the reaction was vigorously stirred in front of white LED while cooling with compressed air to maintain ambient temperature. The ampule was taken out from the white LED irradiation at different times to stop the polymerization. After centrifugation, the BaTiO_3_-based nanoparticles were removed, and the as-prepared solution was diluted by acetone, and then precipitated into the mixed solvent (methanol/water, v/v = 1/1). After filtration, the free PMMA polymers were purified via dissolution/precipitation twice by acetone and methanol/water, and then dried at 50 °C in vacuum for 12 h.

### Detachment of PMMA chains from the surface of BaTiO_3_ nanoparticles for measuring the molecular weight of PMMA grafting chains

PMMA polymers as grafting chains on the surface of BaTiO_3_ nanoparticles were detached from the surface of BaTiO_3_ nanoparticles by dispersing core/shell tetragonal BaTiO_3_/PMMA hybrid nanoparticles in the pyridine: 0.2 g core/shell BaTiO_3_/PMMA hybrid nanoparticles (sample in Fig. 5) were dissolved into 50 mL of pyridine. The mixture solution was stirred at 100 °C for 24 h. After the reaction, the resulting BaTiO_3_ without PMMA was gradually precipitated in pyridine. After filtration, the resulting solution was concentrated to dryness, and then the polymers were dissolved in acetone, and following by precipitating in the mixed solvent (methanol/water, v/v = 1/1). The PMMA polymers can be purified by dissolution/precipitation twice with acetone and methanol/water, and then dried at 50 °C in vacuum for 12 h.

## Results and Discussion

**Figure 2:**
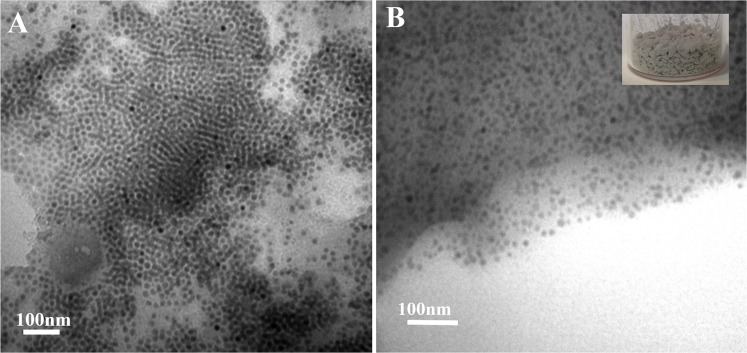
TEM images of cubic BaTiO_3_ nanoparticles embedded in HBPA matrix with different scale bars. The inset in (**B**) shows the digital image of BaTiO_3_/HBPA sample.

As shown in Figs 1 and S1, hyperbranched polyamide (HBPA) with benzimidazole rings was synthesized through a one-step procedure with equimolar monomers of trimesic acid and 2-(4-aminophenyl)-1H-benzimidazol-5-amine. The whole polycondensation process was easily conducted in a homogeneous solution^32^. Moreover, it is noteworthy that the used solvent during the polymerization process can make Ba(OH)_2_ and TTIP (precursors of BaTiO_3_ nanoparticles) dissolved. The reaction solution was slowly heated to 90 °C and refluxed for 3 h. After that, Ba(OH)_2_ (10 mmol) and TTIP (10 mmol), as precursors, were added into the reaction solution (the molar ratio of precursors to benzimidazole ring = 1:1). After another 1 h reaction, the pale yellow precipitates of cubic BaTiO_3_/HBPA were formed, and cubic BaTiO_3_ nanoparticles were encapsulated into the HBPA functional matrix. The chemical structure of HBPA was confirmed by FT-IR, ^1^H and ^13^C-NMR analyses, and the characterization results were shown in Figs S2–S4, respectively. Firstly, SEM characterization was used to the morphology of cubic BaTiO_3_/HBPA nanocomposites, and Fig. S6 show the SEM images of the sample. Clearly, nonuniform submicrospheres were observed. In contrast, the internal structures of cubic BaTiO_3_/HBPA nanocomposites were investigated and confirmed by TEM characterization. The dark dots appearing in the TEM images referred to BaTiO_3_ nanoparticles in HBPA matrix (the average diameter: 18.1 ± 1.9 nm) shown in Fig. 2. Besides, X-ray diffraction (XRD) measurement was applied to characterize the crystalline phase architecture of BaTiO_3_/HBPA nanocomposites, the diffraction pattern shown in Figs S7 and S9(A). According to XRD patterns (patterns with 2θ = 20–60° are shown in Fig. S7; patterns with 2θ = 43–47° are shown in Fig. S9(A)), a single peak with 2θ at around 43–47° ((200) lattice plane) can be observed, suggesting the cubic crystalline phase of BaTiO_3_ nanoparticles^13^.

**Figure 3:**
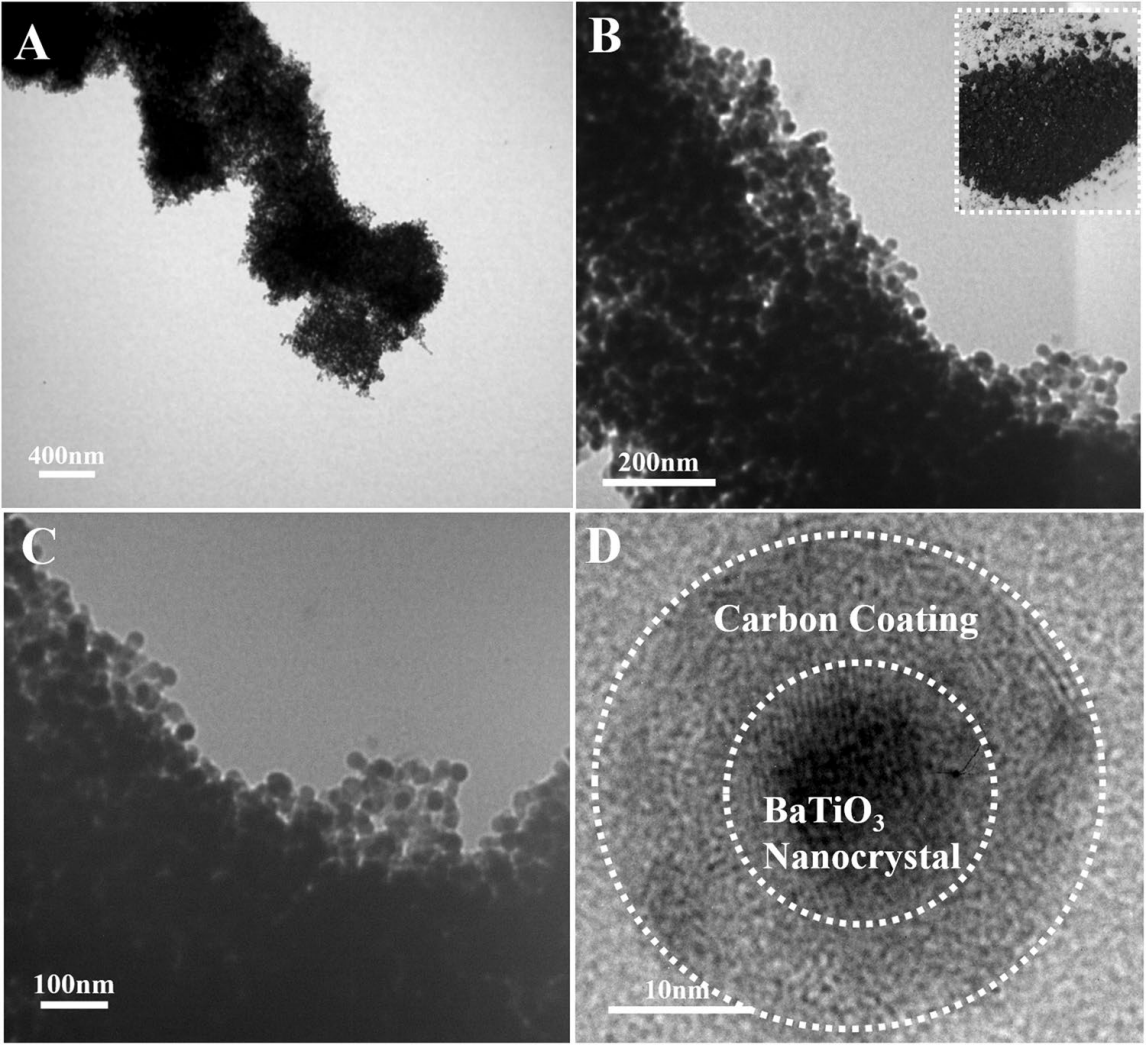
Representative TEM images of tetragonal BaTiO_3_ after high temperature calcinations. (**A**,**B,C**) TEM images of tetragonal BaTiO_3_ nanocrystals coated with carbon layer. (**D**) HR-TEM image of tetragonal BaTiO_3_ nanoparticles capped with carbon layer. The digital image of tetragonal BaTiO_3_ nanoparticles capped with carbon layer as the inset in (**B**).

In Fig. 1, the HBPA coating layer of cubic BaTiO_3_ nanoparticles was easily carbonized under an argon (Ar) at 1200 °C to form a carbon coating layer as protecting shell of the BaTiO_3_ nanoparticles, thereby preventing BaTiO_3_ nanoparticles from aggregation to form irregular larger structures. Meanwhile, after the high temperature calcination, crystalline structure of BaTiO_3_ nanoparticles changed from cubic to tetragonal BaTiO_3_ nanocrystals. The morphology of tetragonal BaTiO_3_ nanocrystals capped by carbon layer was observed from the TEM images in Fig. 3. By comparing TEM characterization results of the BaTiO_3_ nanoparticles before and after high temperature calcination, it was confirmed that dimension distribution of the nanoparticles was non-uniform due to the irregular carbon coating. The architecture of the carbon-coated BaTiO_3_ nanocrystals can be also more clearly observed by high resolution TEM (HR-TEM). Clearly, the crystalline lattices of tetragonal BaTiO_3_ nanoparticles were shown in inner dashed circle of Fig. 3(D), and irregular amorphous carbon coating was shown in outer dashed circle. TEM images indicate that the average diameter of tetragonal BaTiO_3_ nanocrystals including outer irregular carbon coating is about 18 nm. Furthermore, in order to confirm the crystal architecture of tetragonal BaTiO_3_ nanoparticles, X-ray diffraction (XRD) measurement is also carried out shown in Figs S8 and S9(B). XRD curves clearly show strong diffraction peaks. The single peak (2θ = 43–47°) splits into two peaks^33,34^. Detailed refinement of crystal architecture indicates that these as-prepared BaTiO_3_ nanoparticles are largely tetragonal, including some detectable orthorhombic phase. In addition, energy dispersive spectroscopy (EDS) analysis characterization was also conducted to determine the composition of tetragonal BaTiO_3_ nanoparticles capped by carbon layer (in Fig. S10).

**Figure 4:**
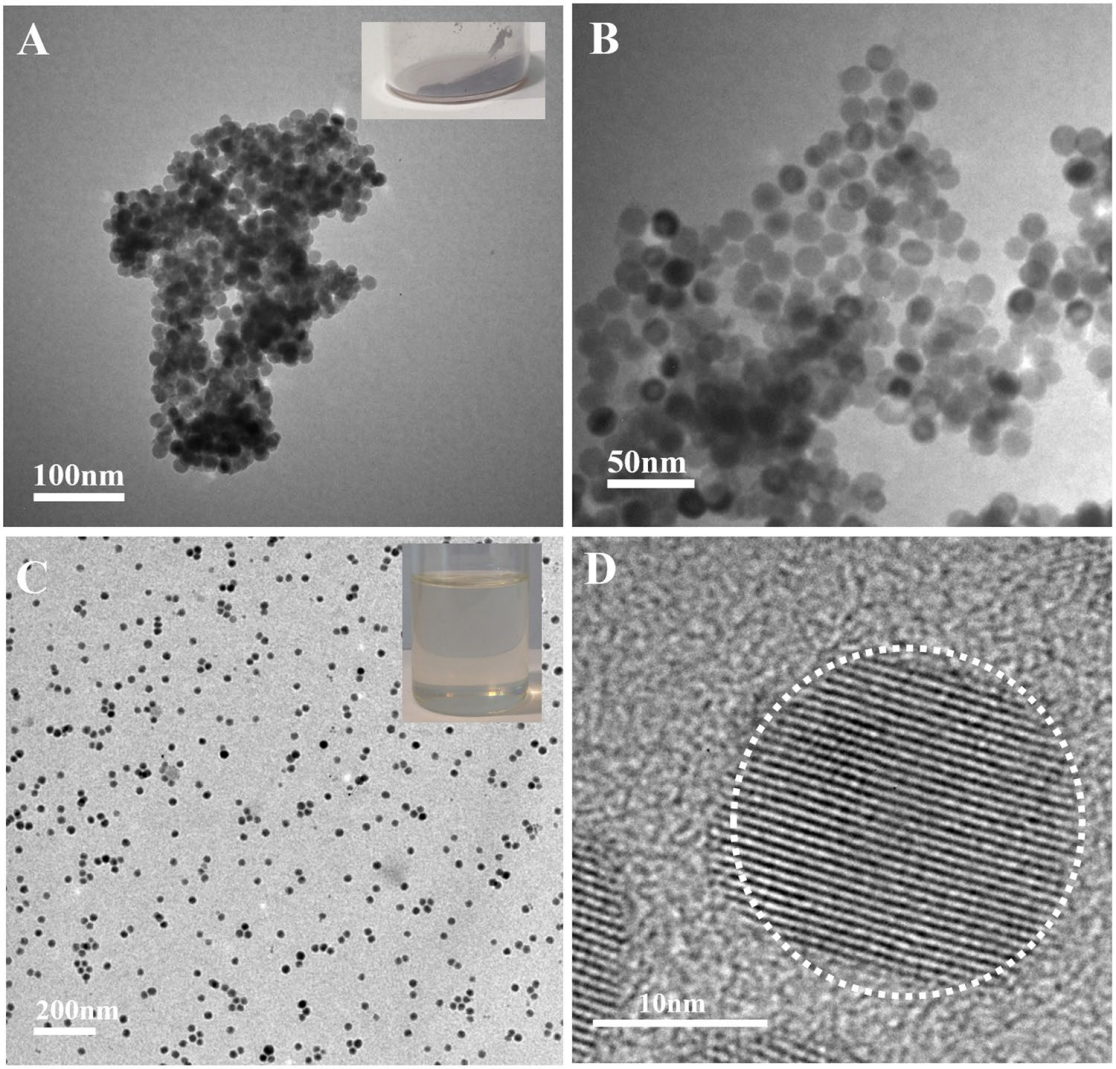
Representative TEM images tetragonal BaTiO_3_ nanoparticles. (**A,B**) TEM images of tetragonal BaTiO_3_ nanoparticles after the removal of carbon shell layer with different scale bars. (**C**) TEM image of tetragonal BaTiO_3_ nanoparticles capped by bi-functional ligands. (**D**) HR-TEM of tetragonal BaTiO_3_ nanocrystals coated with bi-functional ligands. The digital image of tetragonal BaTiO_3_ nanoparticles without carbon layer as the inset in (**A**). The digital image of toluene solution of tetragonal BaTiO_3_ nanocrystals coated with bi-functional ligands as the inset in (**C**).

The carbon coating of tetragonal BaTiO_3_ nanoparticles needs to be removed prior to preparation of the final core/shell tetragonal BaTiO_3_/HBPA nanoparticles by relative low temperature calcination under air (500 °C). Owing to the thermodynamic stability, the shapes of the tetragonal BaTiO_3_ nanocrystals can be almost kept after 500 °C calcination in air^14^. After removing the carbon coating layer, the color of tetragonal BaTiO_3_ nanocrystals powder turned into gray which was shown as insets of Fig. 4(A,B). The tetragonal BaTiO_3_ nanocrystals were characterized by TEM after removal of carbon coating (Fig. 4(A,B)) to compare the morphology under different conditions. According to the TEM images, nanocrystals with about 17 nm of the average diameter are uniform. In addition, the composition of nanocrystals can be further determined by EDS microanalysis characterization after removing of carbon layer (Fig. S11). On the basis of the EDS characterization, it was found that the carbon shell layer was almost completely disappeared after 500 °C calcination under air for 5 h.

Surface modification of these tetragonal BaTiO_3_ nanocrystals without carbon coating layer is necessary to prepare tetragonal BaTiO_3_ nanoparticles coated with a metal-free ATRP initiators, the whole process is depicted in Fig. 1. The tetragonal BaTiO_3_ nanoparticles were dispersed in toluene by ultrasonication process, followed by the addition of bifunctional ligands to the toluene solution. Owing to the coordination bond interaction between carboxyl group of the bifunctional ligands and surface metal atom on tetragonal BaTiO_3_ nanoparticles, tetragonal BaTiO_3_ nanoparticles covered with metal-free ATRP initiating sites were formed by tethering bi-functional ligands onto the surface of nanoparticles, which showed compact comb-like structure^35–38^. BaTiO_3_ nanocrystals without any ligands were easily precipitated in organic solvent (e.g., toluene), while tetragonal BaTiO_3_ nanoparticles coated with metal-free ATRP initiating sites can be easily dissolved in organic solvent (e.g., toluene) (Fig. 4(C)). The morphology of tetragonal BaTiO_3_/bifunctional ligand nanocrystals was investigated by TEM measurements, and the representative TEM and HR-TEM images are shown in Fig. 4(C,D). It is confirmed by analyzing TEM images that the average size of tetragonal BaTiO_3_ nanocrystals is 17.1 ± 1.9 nm, smaller than the average diameter of cubic BaTiO_3_ nanoparticles because of the crystalline transformation and perfection. For the determination of the presence of metal-free ATRP initiators on the surface of tetragonal BaTiO_3_, TGA was also applied (Fig. S12(A)). The weight fraction of metal-free ATRP initiators is confirmed to be 4.6% (the areal density of initiators on the surface of BaTiO_3_ nanoparticles: 2.09/nm^2^). For investigating stability of bi-functional ligands on the surface of BaTiO_3_ nanoparticles in organic solvent (e.g., toluene), toluene solution of BaTiO_3_ nanoparticles coated with bi-functional ligands was vigorously stirred for 48 h at room temperature. After BaTiO_3_ nanoparticles coated with bi-functional ligands were obtained by centrifuging, no bi-functional ligand was detected in residual solution. In addition, TGA curve (Fig. S12(B)) is almost same with freshly prepared sample (almost same weight fraction of the bi-functional ligands). These experimental results suggest that the desorption of bi-functional ligands doesn’t happen when dispersing the BaTiO_3_/bi-functional ligands in solvent.

**Figure 5:**
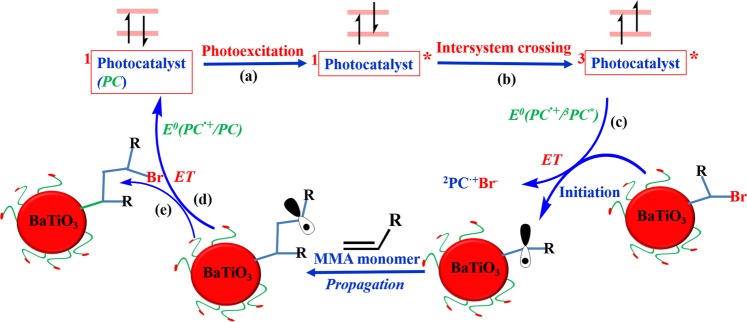
A proposed mechanism of metal-free ATRP mediated by a photocatalyst (PC) via (**A**) photoexcitation to ^1^PC^*^; (**B**) intersystem crossing (ISC) to the triplet state (^3^PC^*^); (**C**) ET to form the radical cation doublet (^2^PC^*•*+^); (**D**) back ET to regenerate PC; (**E**) reversibly terminate monomer polymerization.

Thereafter, the metal-free ATRP initiators on the surface of tetragonal BaTiO_3_ nanoparticles were utilized to initiate the polymerization of MMA monomers under a white LED irradiation at ambient temperature for the preparation of core/shell tetragonal BaTiO_3_/PMMA nanoparticles^27^. During the metal-free ATRP process, 5,10-di(1-naphthyl)-5,10-dihydrophenazine was used as an organic photocatalyst (PC). Our proposed initiating mechanism of MMA monomers postulates reversible electron transfer (ET) from the photoexcited PC for reversibly activating an alkyl bromide initiator (Fig. 5)^27^. Except for the requirement of the excited triplet state (^3^PC^*^) with sufficiently strong reducing ability to activate the ATRP initiating sites, it is necessary that an interplay should be balanced between the ability to oxidize the propagating radical and its stability of the radical cation (^2^PC^•+^) in order to efficiently deactivate the propagating polymeric chain and yield a controlled radical polymerization^26^. Based on computationally guided discovery^39,40^, we choose 5,10-di(1-naphthyl)-5,10-dihydrophenazine as a PC for the fabrication of tetragonal BaTiO_3_/PMMA core/shell nanoparticles. It is worth noting that the phenazine core was shared by several different biologically relevant molecules used as redox-active antibiotics^41^, whereas phenazine-based derivatives have attracted considerable attention in the field of organic photovoltaics^42,43^.

**Figure 6:**
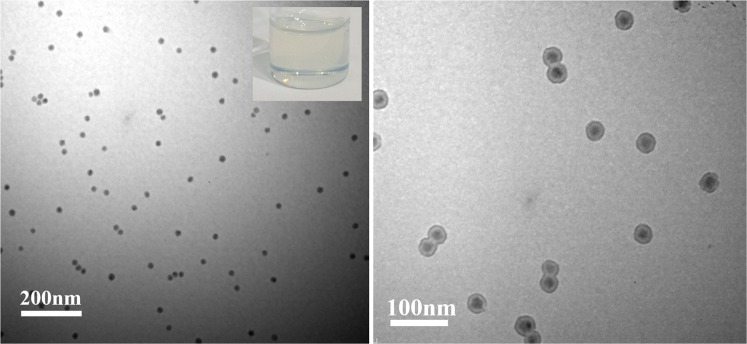
TEM characterization of core/shell tetragonal BaTiO_3_/PMMA nanoparticles with different scale bars. (**A**) TEM image of core/shell tetragonal BaTiO_3_/PMMA nanoparticles; Inset: digital image of toluene solution of tetragonal BaTiO_3_/PMMA nanoparticles. (**B**) TEM image of core/shell tetragonal BaTiO_3_/PMMA nanoparticles after PMMA macromolecular shell stained by RuO_4_.

Although 5,10-di(1-naphthyl)-5,10-dihydrophenazine as a photocatalyst has been investigated for the organocatalyzed radical polymerizations^27^, for the first time, metal-free ATRP driven by visible light by using 5,10-di(1-naphthyl)-5,10-dihydrophenazine as photocatalyst was applied for the fabrication of size-tunable core/shell ferroelectric/polymeric nanoparticles. During the conventional ATRP process, a variety of ligated metal catalysts, such as Cu(I), Fe(II), Ru(II) and so on, were usually utilized to mediate the equilibrium process of the redox. Nevertheless, for the fabrication of functional inorganic/organic nanocomposites by ATRP techniques, a crucial limiting factor is the contamination and purification of metal catalysts. In our fabrication approach, the PMMA shell thickness can be readily controlled by tuning the white LED irradiation time during the metal-free ATRP process (Fig. S13). Firstly, the tetragonal BaTiO_3_/bi-functional ligands nanoparticles (the average diameter: 17.1 ± 1.9 nm) were utilized as example, PMMA chains as polymeric shell can grow from the surface of tetragonal BaTiO_3_ nanocrystals, 5,10-di(1-naphthyl)-5,10-dihydrophenazine as the ATRP photocatalyst under white LED irradiation at ambient temperature (5 h). The architectures of core/shell tetragonal BaTiO_3_/PMMA nanoparticles can be further investigated using TEM measurement by staining the PMMA chains of polymeric shell with ruthenium tetraoxide (RuO_4_)^44–46^. The clear ~6 nm shell thickness in the TEM images corresponding to PMMA chains can be investigated (Fig. 6). In addition, for obtaining real information of PMMA grafting chains, the detaching of PMMA shell from the tetragonal BaTiO_3_ nanocrystal surface was carried out by dispersing core/shell tetragonal BaTiO_3_/PMMA hybrid nanoparticles in the pyridine. According to GPC characterization, a single peak with narrow distribution (PDI: 1.25) can be observed for the detached polymeric chains (Fig. S14). In addition, the molecular weight of 11,300 g/mol is close to that of PMMA polymers synthesized from free ATRP initiators (12,950 g/mol). It is worth noting that the existence of BaTiO_3_ core has almost no effect on the metal-free ATRP process of MMA monomers. By analyzing ^1^H-NMR and TGA characterization results, it was confirmed that PMMA polymeric shell existed on the surface of BaTiO_3_ core (Figs S15 and S16). The weight fraction of total organic shell (initiators and PMMA) was determined by TGA (18.4%), and the weight fraction of PMMA shell was 13.8%.

**Figure 7:**
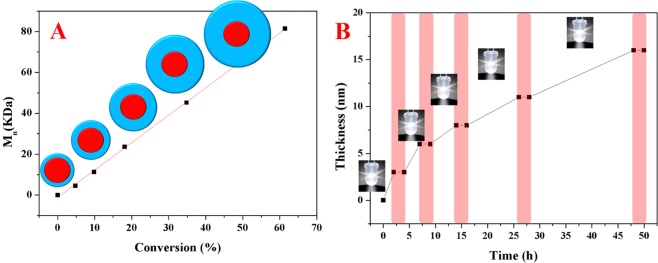
Results for the polymerization of MMA by metal-free ATRP driven by visible light. (**A**) Plot of Mn versus monomer conversion for the metal-free ATRP of MMA monomers under continuous irradiation (the detachment of PMMA chains from the surface of tetragonal BaTiO_3_ nanocrystals). (**B**) Plot of thickness of PMMA polymeric shell versus irradiation time during pulsed light irradiation with white LEDs.

The PMMA shell thickness can be readily controlled by tuning the white LED irradiation time during the metal-free ATRP, and all results about PMMA shell are summarized in Table S1 and Table S2. For example, with the white LED irradiation time increasing to 20 h, the PMMA shell thickness can be tuned to ~11 nm. In addition, temporal control has been realized by utilizing a pulsed-irradiation sequence (Fig. 7). The PMMA shell thickness increasing driven by visible light has been only detected during irradiation, and the increasing paused during dark time. At the same time, the corresponding molecular weight of PMMA chains steadily increased with the irradiation time increasing (Fig. 7(A) and Table S2). Importantly, the dimensions of tetragonal BaTiO_3_ nanoparticles can be tuned by adjusting the molar ratio of benzimidazole ring units to precursors during the polycondensation process (Table S3 and Fig. S17). The size increase of nanocrystals may be the result of the higher precursor concentration in reaction solution^29,47^. For instance, when the molar ratio of precursors to benzimidazole ring units was adjusted from 1:1 to 10:1, the average size of tetragonal BaTiO_3_ nanoparticles could be tuned to 39.2 ± 4.2 nm as other conditions fixed (Fig. S18). In order to further confirm that sizes of BaTiO_3_ nanoparticles are independent on ATRP process of MMA monomers, larger BaTiO_3_ nanoparticles (*D*: ~39 nm, Sample-5 in Table S3) capped with bi-functional ligands were used as initiators to initiate polymerization of MMA monomers under different LED irradiation time. Comparing with smaller BaTiO_3_ nanoparticles (samples in Table S1), the thickness of shell is almost same under same LED irradiation time (samples in Table S4).

**Figure 8:**
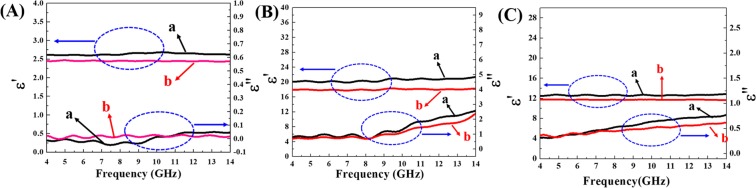
The dielectric properties of core/shell tetragonal BaTiO_3_/PMMA hybrid nanoparticles and corresponding PMMA shell (frequency range: 4–14 GHz). (**A**) The PMMA shell with low molecular weight (a; Sample-2 in Table S2, *M*_*n*,*GPC*_ = 11.3 *KDa*) and high molecular weight (b; Sample-3 in Table S2, *M*_*n*,*GPC*_ = 23.4 *KDa*); (**B**) The core/shell tetragonal BaTiO_3_/PMMA hybrid nanoparticles with same shell size (Sample-2 in Table S2, *M*_*n*,*GPC*_ = 11.3 *KDa*) and different sizes of core BaTiO_3_ nanoparticles (~17 nm (b) and ~39 nm (a); Sample-1 and Sample-5 in Table S3); (**C**) The core/shell tetragonal BaTiO_3_/PMMA hybrid nanoparticles with same shell size(Sample-3 in Table S2, *M*_*n*,*GPC*_ = 23.4 *KDa*) and different sizes of core BaTiO_3_ nanoparticles (~17 nm (b) and ~39 nm (a); Sample-1 and Sample-5 in Table S3).

The dielectric properties of core/shell tetragonal BaTiO_3_/PMMA hybrid nanoparticles and corresponding PMMA shell are shown in Fig. 8 (the frequency range: *f* = 4–14 GHz). For example, the real part of permittivity (ε′ = 2.71 ± 0.52) of the PMMA shell with low molecular weight (Sample-2 in Table S2, *M*_*n*,*GPC*_ = 11.3 *KDa*) is larger than that of the sample with high molecular weight (Sample-3 in Table S2, *M*_*n*,*GPC*_ = 23.4 *KDa*) (ε′ = 2.41 ± 0.34). Due to the higher coiling degree of the longer polymeric chains, the value of ε′ decreases with increase of *M*_*n*,*GPC*_ of PMMA shell^48^. The imaginary part values of permittivity (ε″) of two samples are almost zero, and indicated that the dielectric properties are nearly lossless (Fig. 8(A)). The permittivity values of core/shell tetragonal BaTiO_3_/PMMA hybrid nanoparticles with same shell size (Sample-2 in Table S2, *M*_*n*,*GPC*_ = 11.3 *KDa*) and different sizes of core BaTiO_3_ nanoparticles (~17 nm and ~39 nm; Sample-1 and Sample-5 in Table S3) are shown in Fig. 8(B). The real part of permittivity ε′ (22.23 ± 1.09) of core/shell BaTiO_3_/PMMA hybrid nanoparticles (core: ~39 nm) is larger than that (17.06 ± 0.58) of sample (core: ~17 nm). At the same time, the permittivity values of core/shell BaTiO_3_/PMMA hybrid nanoparticles fabricated by same core BaTiO_3_ nanoparticles and different PMMA shell molecular weights (Sample-3 in Table S2, *M*_*n*,*GPC*_ = 23.4 *KDa*) are shown in Fig. 8(C). In contrast, it is clear that both ε′ and ε″ are lower than the case of the lower molecular weight PMMA as shell. The dielectric property difference between different core/shell BaTiO_3_/PMMA hybrid nanoparticles (Fig. 8(B,C)) composed of same BaTiO_3_ core may be attributed to the different molecular weights of PMMA shell and different volume fractions of core nanoparticles from different thickness of PMMA shell. In addition, the difference of dielectric properties of different core/shell BaTiO_3_/PMMA hybrid nanoparticles composed of same PMMA shell thickness can be due to the size effect of core BaTiO_3_ nanocrystals^17,49–51^. In general, the results in Fig. 8 indicated that the dielectric properties of core/shell BaTiO_3_/PMMA hybrid nanoparticles can be tuned by adjusting the dimension of BaTiO_3_ core and the molecular weight of PMMA shell.

## Conclusion

In conclusion, an unconventional but facile approach to prepare size-tunable core/shell ferroelectric/polymeric nanoparticles with uniform distribution was reported by metal-free ATRP driven by visible light under ambient temperature based on novel HBPA as functional matrix. Cubic BaTiO_3_/HBPA nanocomposites, HBPA as matrix and Ba(OH)_2_ and TTIP as precursors, were firstly fabricated by *in-situ* direct polycondensation process. In an inert atmosphere, the aromatic polyamide as capping layer of cubic BaTiO_3_ nanocrystals was readily carbonized by 1200 °C calcination to form carbon coating layer on the surface of BaTiO_3_ nanoparticles for preventing these nanocrystals from aggregation and merging. The cubic BaTiO_3_ nanocrystals were simultaneously transformed into tetragonal BaTiO_3_ after 1200 °C calcination. Then the outer carbon layer as shell coating of tetragonal BaTiO_3_ nanoparticles can be removed via the calcination under relative low temperature (500 °C) in air. Then the bi-functional ligands were used for the surface modification of tetragonal BaTiO_3_ nanocrystals. PMMA polymeric chains were growing from the initiating sites of ferroelectric BaTiO_3_ nanocrystal surface by the metal-free ATRP technique to obtain core/shell ferroelectric BaTiO_3_/PMMA hybrid nanoparticles, composed of ferroelectric BaTiO_3_ nanocrystals as core and PMMA polymeric chains as shell. The size of ferroelectric BaTiO_3_ nanoparticles can be tuned when the molar ratio of benzimidazole ring units to precursors was changed in the polycondensation process, and the thickness of polymeric shell can be also tailored by changing the white LED irradiation time within the organocatalyzed ATRP process. The dielectric properties of core/shell BaTiO_3_/PMMA hybrid nanoparticles can be tuned by adjusting the dimension of BaTiO_3_ core and the molecular weight of PMMA shell. Therefore, we envisage that this facile approach based on metal-free ATRP driven by visible light at ambient temperature would open up a new avenue for producing a variety of intriguing novel functional organic/inorganic hybrid nanomaterials for many applications (e.g., catalysts, electronics, etc.).

## Supplementary information

Supporting Information
