# Supplementary material for: Facile Fabrication of Size-Tunable Core/Shell Ferroelectric/Polymeric Nanoparticles with Tailorable Dielectric Properties via Organocatalyzed Atom Transfer Radical Polymerization Driven by Visible Light
Source: Sci Rep. 2019 Feb 12;9:1869. doi: 10.1038/s41598-018-38039-8 (PMC6372659; doi:10.1038/s41598-018-38039-8)
Supplement: Supplementary file 1 — Supporting Information [file 41598_2018_38039_MOESM1_ESM.docx]

**Supporting Information:**

**Facile Fabrication of Size-Tunable Core/Shell Ferroelectric/Polymeric Nanoparticles** **with Tailorable Dielectric Properties via Organocatalyzed Atom Transfer Radical Polymerization Driven by Visible Light**

*Ning You ^1,2,3§^, Chenxi Zhang^1,2,3§^, Yachao Liang^1,2,3^, Qi Zhang^1,2,3^, Peng Fu^1,2,3^, Minying Liu^1,2,3^, Qingxiang Zhao^1,2,3^, Zhe Cui^1,2,3^* &Xinchang Pang^1,2,3^**

^1^School of Materials Science and Engineering, Zhengzhou University, Zhengzhou 450001, China

^2^ Engineering Laboratory of High Performance Nylon Engineering Plastics of CPCIF, Zhengzhou University, Zhengzhou 450001, China

^3^Henan Joint International Research Laboratory of Living Polymerizations and Functional Nanomaterials, Zhengzhou University, Zhengzhou 450001, China

*To whom correspondence should be addressed. ^§^These authors contributed equally to this work.

E-mail: cuizhezzu@126.com; [pangxinchang1980@163.com](mailto:pangxinchang1980@163.com)


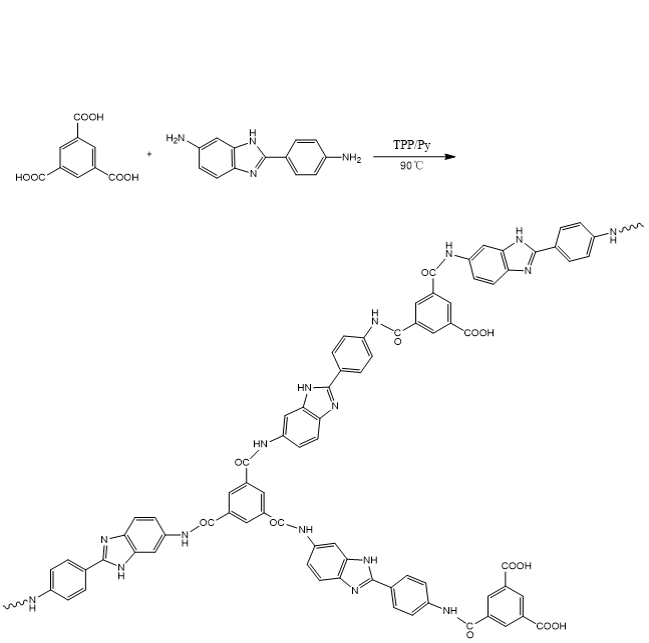


**Figure S1.** The synthetic route of HBPA.


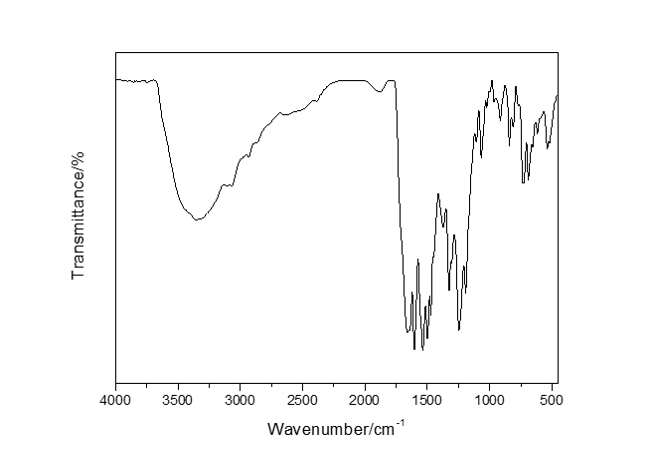


**Figure S2.** FT-IR spectrum of cubic BaTiO_3_/HBPA nanocomposites.

FT-IR spectrum of cubic BaTiO_3_/HBPA nanocomposites was shown in **Figure S2**. All the characteristic peaks of amide groups and carboxy groups of HBPA are listed as follows: 1658 cm^-1^(carboxy, C=O stretching vibration), 1370 cm^-1^ (carboxy, C-O stretching vibration), 1640 cm^-1^(amide I, C=O stretching vibration), 1535 cm^-1^(amide II, C-N stretching and CO-N-H bending vibration), 3352 cm^-1^(hydrogen-bonded, N-H stretching vibration and O-H stretching vibration).


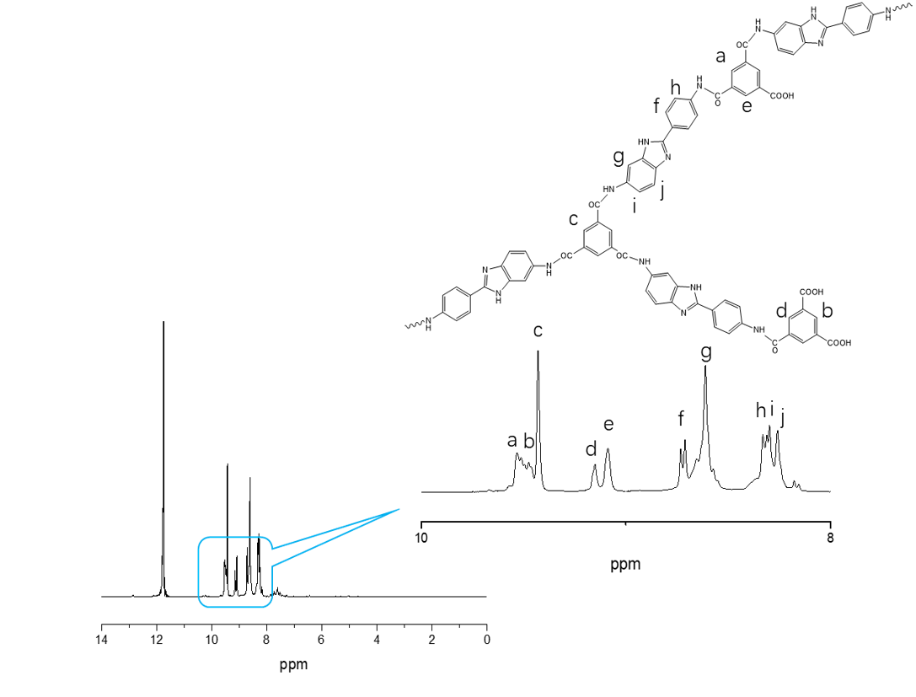


Figure S3. ^1^H-NMR spectrum of cubic BaTiO_3_/HBPA nanocomposites.


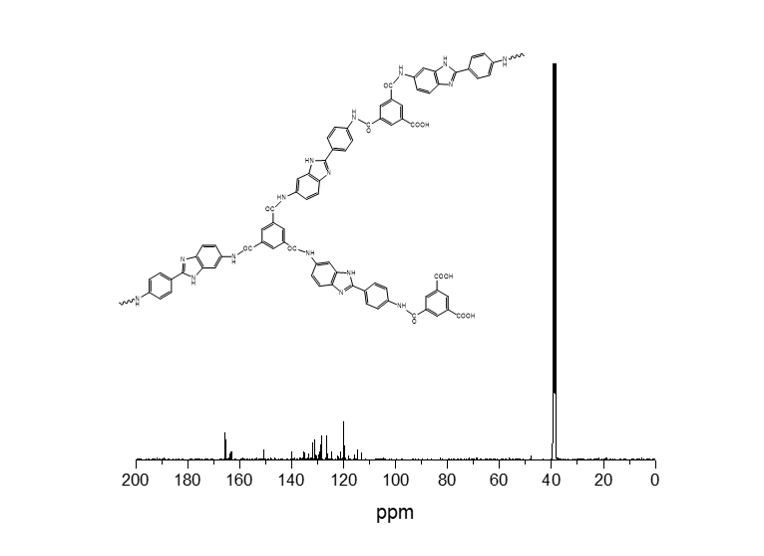


Figure S4. ^13^C-NMR spectrum of cubic BaTiO_3_/HBPA nanocomposites.

**Figure S3** presents the ^1^H-NMR spectrum of cubic BaTiO_3_/HBPA nanocomposites in deuterated trifluoroacetic acid (TFA). The chemical shifts of the protons with different chemical environments were monitored. Multiple peaks attributed to TMA protons were observed from 9.09 to 9.53 ppm. In these peaks, peak at 9.43 ppm (3H) were assigned to the H_c_ protons of the trisubstituted TMA unit, peaks at 9.53 (1H) and 9.09 (2H) ppm was attributed to the H_a_ and H_e_ protons of the bisubstituted TMA unit and the chemical shift at 9.48 (1H) and 9.15 (2H) ppm was assigned to the H_b_ and H_d_ protons of the one-substituted TMA unit. The double peaks at 8.74 (2H) and 8.33 (2H) ppm were assigned to the H_f_ and H_h_ protons of the phenyl ring belonged to diamine unit respectively. The peaks at 8.61 (1H), 8.30 (1H) and 8.26 (1H) ppm were attributed to the H_g_ , H_i_ and H_j_ protons of the benzimidazole ring belonged to diamine units. According to **Figure S4**, all carbon nuclei gave well separated signals in the ^13^C-NMR spectrum.


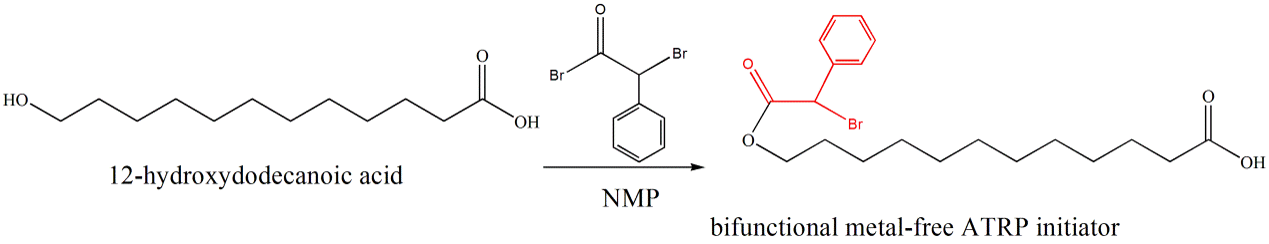


**Figure S5**. Synthesis of bi-functional metal-free ATRP initiators[^1^](#_ENREF_1).


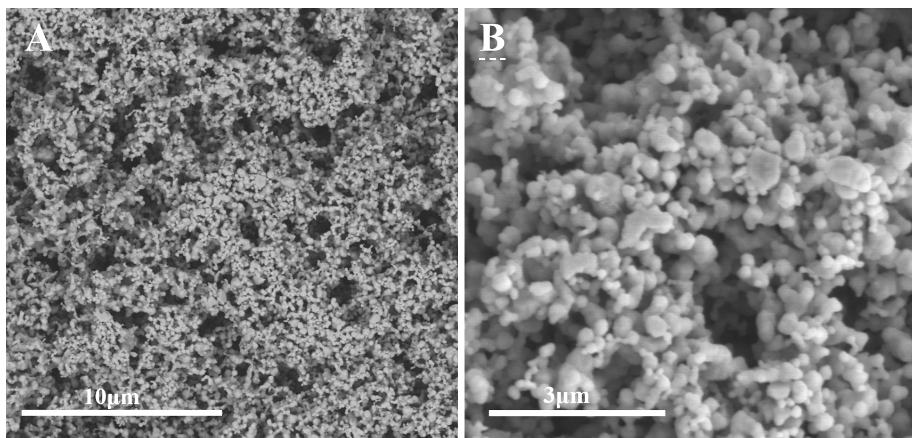


**Figure S6.** SEM images of cubic BaTiO_3_/HBPA nanocomposites with different scale bars: (A) 10 μm; (B) 3μm.


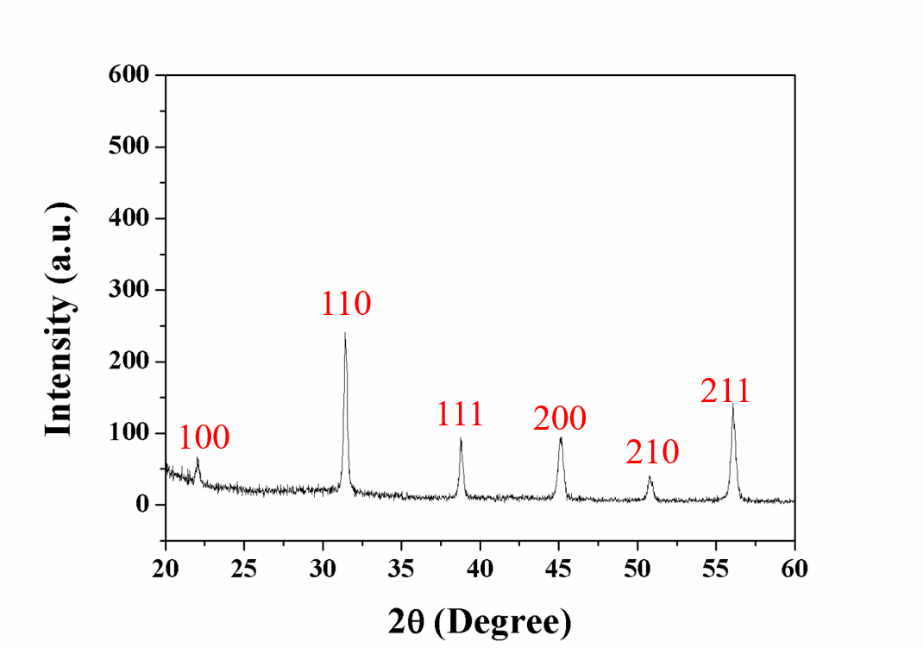


**Figure S7.** XRD pattern of cubic BaTiO_3_/HBPA nanocomposites[^2^](#_ENREF_2).


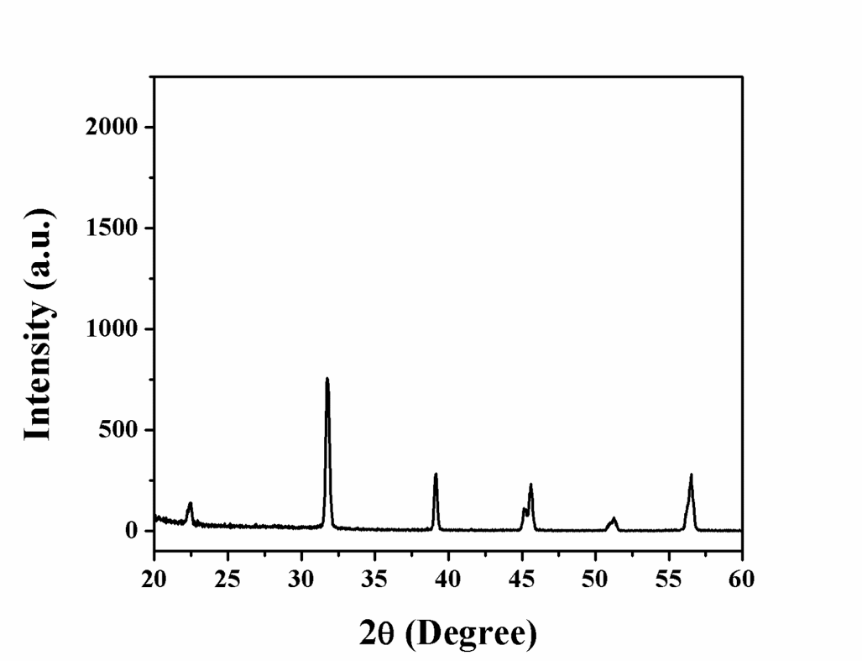


**Figure S8.** XRD pattern of carbon-capped tetragonal BaTiO_3_ nanoparticles[^3^](#_ENREF_3)^,^ [^4^](#_ENREF_4).


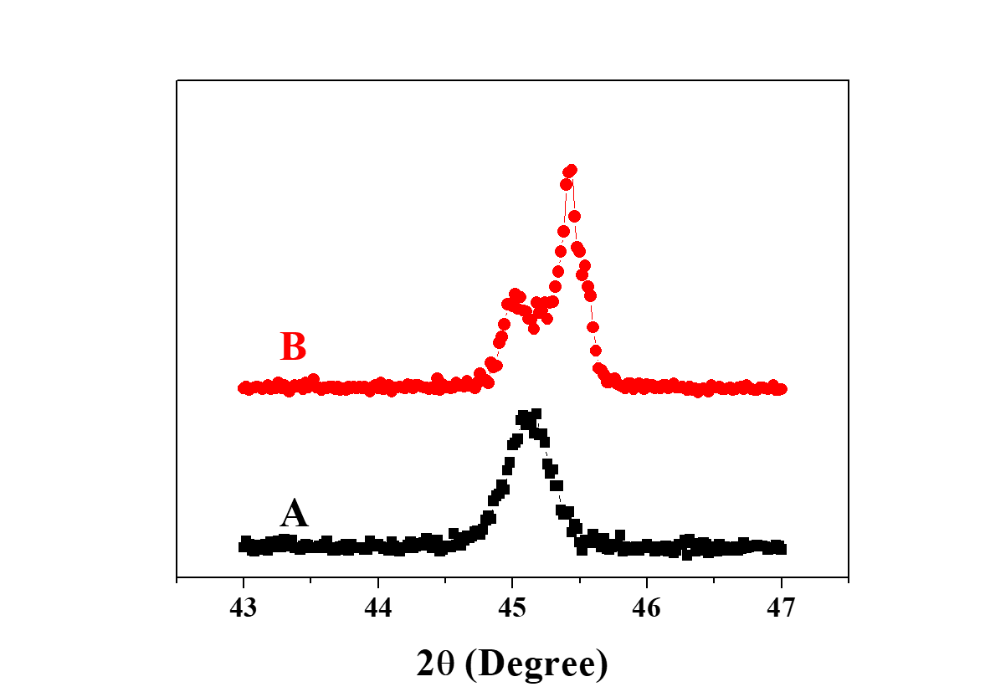


**Figure S9.** XRD patterns of cubic BaTiO_3_/HBPA nanocomposites before and after calcination at 1200^o^C in argon (2h). (A) XRD pattern of cubic BaTiO_3_/HBPA nanocomposites. (B) XRD pattern of carbon-capped tetragonal BaTiO_3_ nanoparticles.


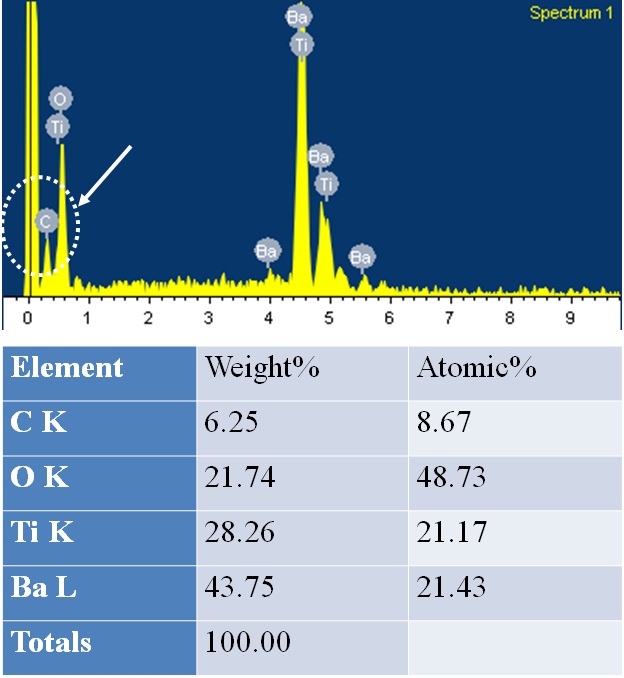


**Figure S10.** EDS spectrum of tetragonal BaTiO_3_ nanocrystals with carbon coating.


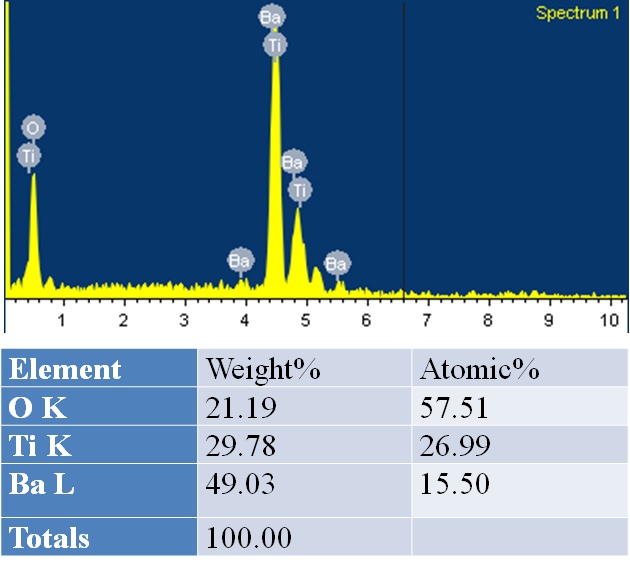


**Figure S11.** EDS spectrum of tetragonal BaTiO_3_ nanocrystals after removing of carbon coating.


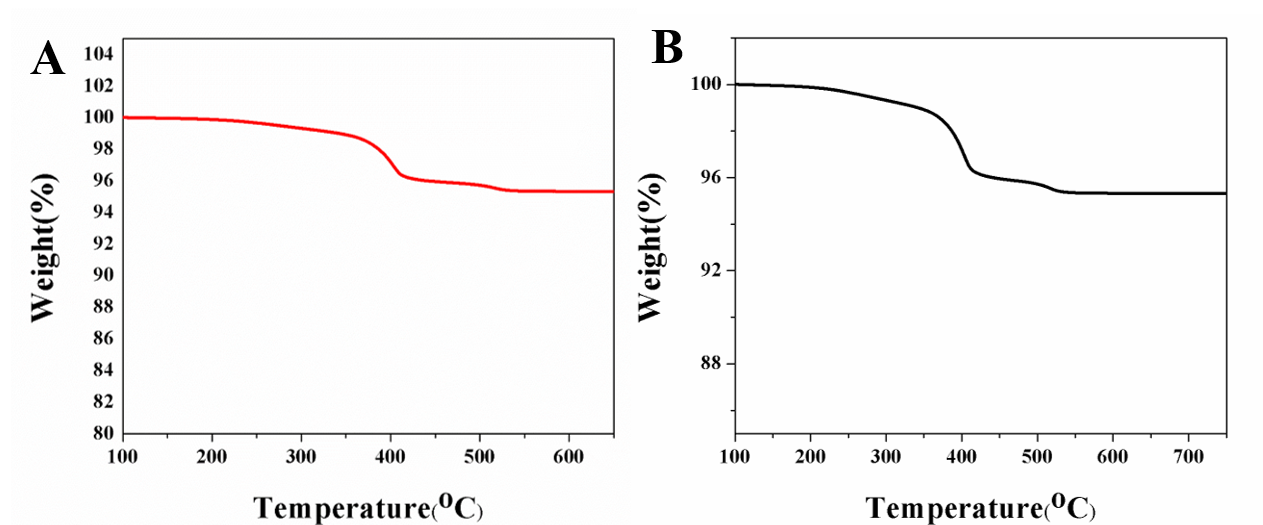


**Figure S12.** TGA curves of tetragonal BaTiO_3_ nanocrystals capped with metal-free ATRP initiators in air (sample in **Figure 4**; heat rate: 10^o^C/min). (A) Freshly prepared sample; (B) Sample collected after dispersion in toluene for 48h to investigate the stability of bi-functional ligands on the surface of BaTiO_3_ nanocrystals.


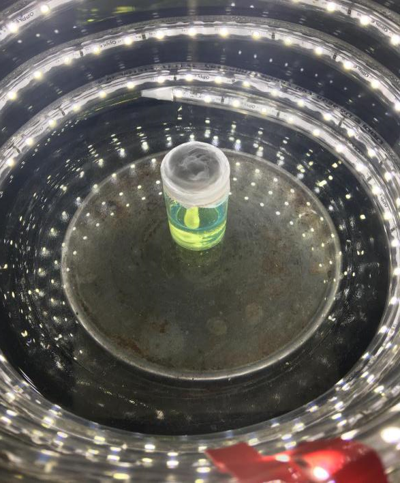


**Figure S13**. Representative configuration composed of reaction vial surrounded by the white LEDs.


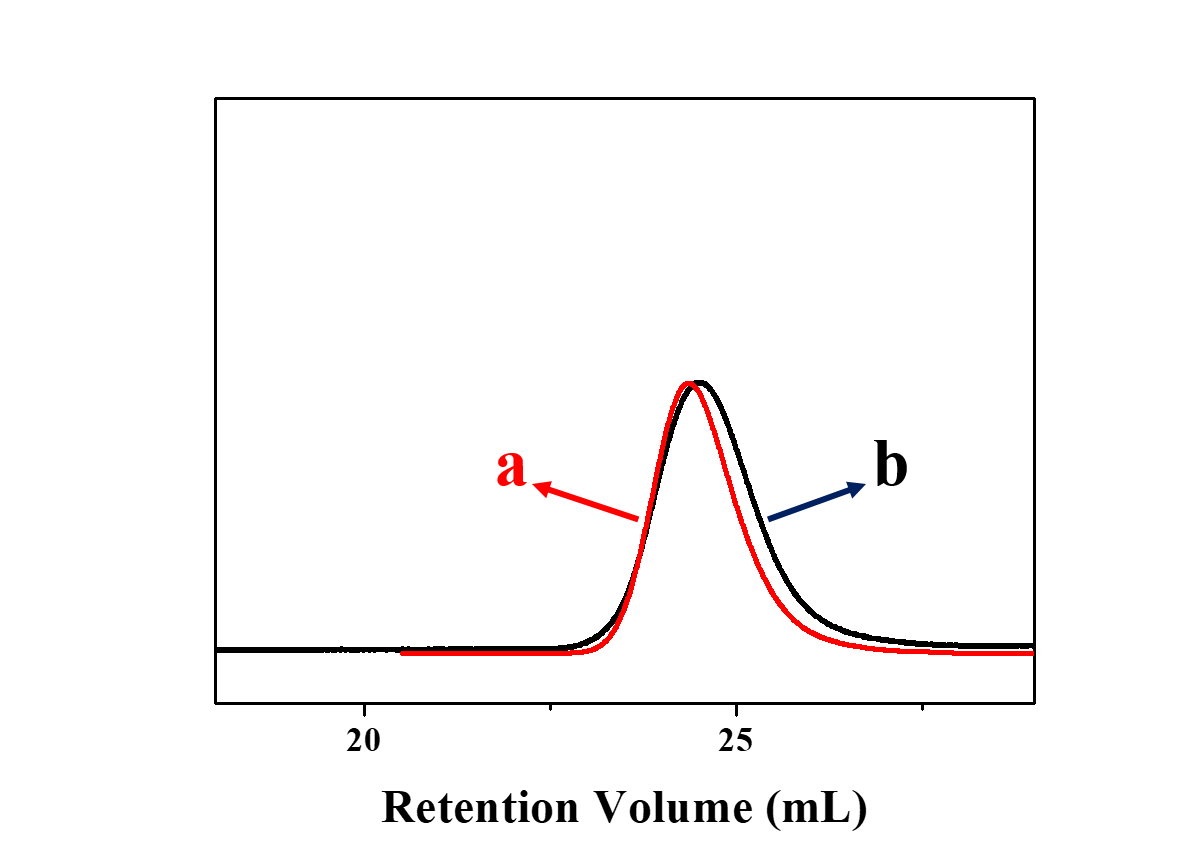


**Figure S14.** GPC traces of PMMA polymeric chains obtained from free initiators (a) and detachment (b) from the surface of tetragonal BaTiO_3_ nanocrystals (sample in **Figure 6**).


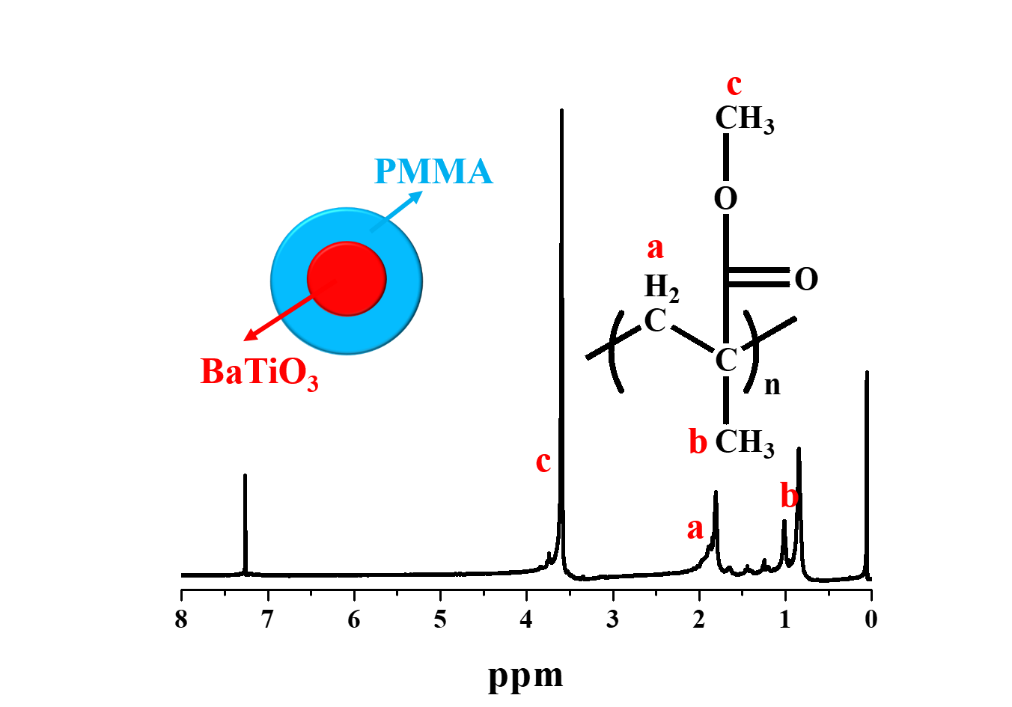


**Figure S15.** ^1^H-NMR spectrum of tetragonal BaTiO_3_ nanocrystals capped with PMMA as shell (core/shell tetragonal BaTiO_3_/PMMA colloidal nanocrystals, solvent: CDCl_3_).


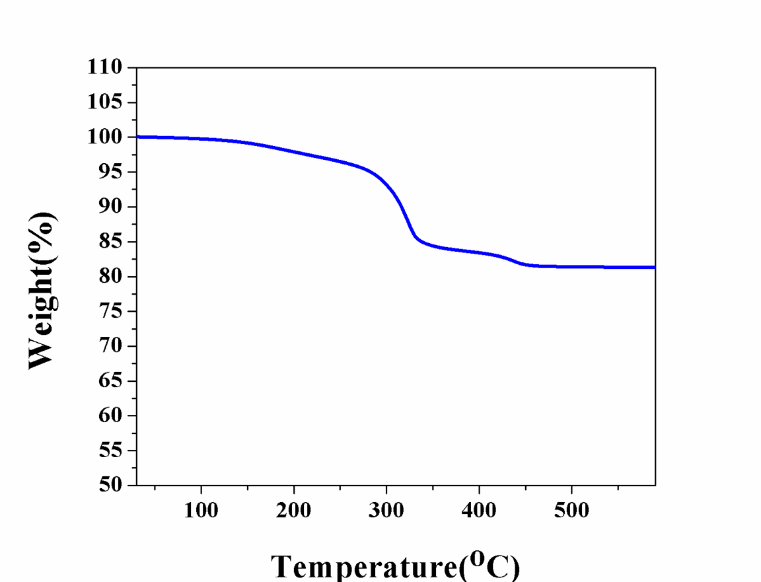


**Figure S16.** TGA curve of tetragonal BaTiO_3_ nanocrystals capped with PMMA as shell (core/shell tetragonal BaTiO_3_/PMMA colloidal nanocrystals) in air (heat rate: 10^o^C/min).

**Table S1.** Summary of core/shell BaTiO_3_/PMMA hybrid nanoparticles

| *Entry^a^* | *Time (h)^b^* | *Thickness (nm)^c^* |
| --- | --- | --- |
| Sample-1 | 2 | 3 |
| Sample-2 | 5 | 6 |
| Sample-3 | 10 | 8 |
| Sample-4 | 20 | 11 |
| Sample-5 | 40 | 16 |

*^a^* Five samples were prepared by tetragonal BaTiO_3_ nanocrystals capped with metal-free ATRP initiators as initiators (sample in **Figure 4(C, D)**). *^b^*The white LED irradiation time at room temperature. *^c^*Thickness of PMMA shell determined by TEM.

**Table S2.** Summary of PMMA polymeric shell detached from the surface of BaTiO_3_ nanoparticles

| *Entry^a^* | *Time (h)^b^* | *Conversion (%)^c^* | *M_n,GPC_*  *(KDa)^d^* | *M_w_/M_n_(PDI)^e^* |
| --- | --- | --- | --- | --- |
| Sample-1 | 2 | 4.9 | 4.5 | 1.26 |
| Sample-2 | 5 | 9.7 | 11.3 | 1.25 |
| Sample-3 | 10 | 18.1 | 23.4 | 1.22 |
| Sample-4 | 20 | 34.9 | 45.1 | 1.20 |
| Sample-5 | 40 | 61.3 | 81.3 | 1.17 |

*^a^*Five samples were prepared by the polymerization of MMA by metal-free ATRP driven by visible light under different irradiation time, tetragonal BaTiO_3_ nanocrystals capped with metal-free ATRP initiators as initiators (sample in **Figure 4(C, D)**). *^b^* White LEDs light irradiation time. *^c^*Determined by gravimetric method. *^d^*Number average molecular weight, *M*_n,GPC_ determined by GPC, calibrated by PS standard. *^e^*Polydispersity index (PDI) measured by GPC.

**Table S3.** Summary of the sizes of tetragonal BaTiO_3_ nanoparticles under the different molar ratios of precursors to benzimidazole ring units

| *Entry ^a^* | *R^b^* | *D(nm) ^c^* |
| --- | --- | --- |
| Sample-1 | 1:1 | 17 |
| Sample-2 | 3:1 | 26 |
| Sample-3 | 5:1 | 32 |
| Sample-4 | 7:1 | 36 |
| Sample-5 | 10:1 | 39 |

*^a^* Five samples were prepared by changing the molar ratio of benzimidazole ring units to precursors during the polycondensation process. *^b^* The molar ratio of precursors to benzimidazole ring units. *^c^* Diameters of tetragonal BaTiO_3_ nanoparticles determined by TEM.





**Figure S17.** Plot of the sizes of tetragonal BaTiO_3_ nanoparticles versus the molar ratio of precursors to benzimidazole ring units.


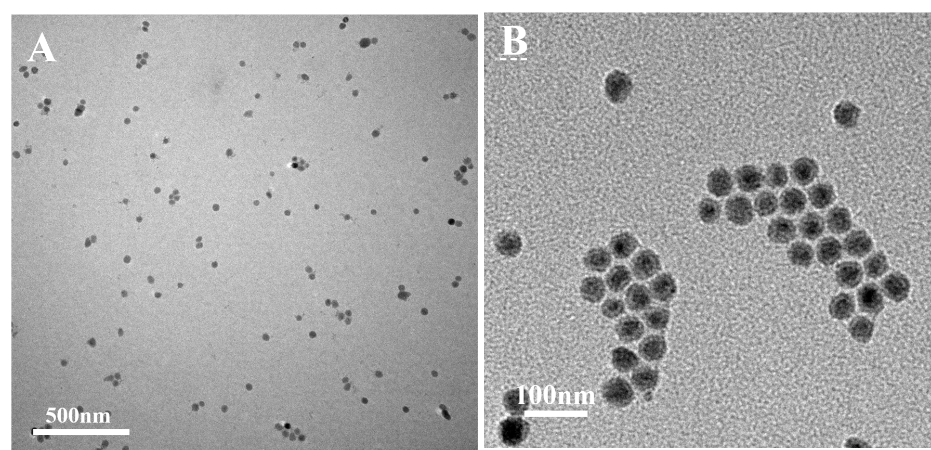


**Figure S18.** Representative TEM image of core/shell tetragonal BaTiO_3_/PMMA colloidal nanocrystals with different scale bars (polymerization of MMA driven by the white LEDs, irradiation time: 5h), core BaTiO_3_ nanocrystals with the average diameter of 39.2±4.2 nm when the molar ratio of precursors to benzimidazole ring units was increased to 10:1 (Sample-5 in **Table S3**). (A) TEM image of core/shell tetragonal BaTiO_3_/PMMA nanoparticles. (B) TEM images of core/shell tetragonal BaTiO_3_/PMMA nanoparticles after PMMA macromvolecular shell stained by RuO_4_.

**Table S4.** Summary of core/shell BaTiO_3_/PMMA hybrid nanoparticles using Sample-5 (*D*: ~39nm) in **Table S3** as core and initiators when other reaction conditions were kept same with samples in **Table S1**

| *Entry^a^* | *Time (h)^b^* | *Thickness (nm)^c^* |
| --- | --- | --- |
| Sample-1 | 2 | ~3 |
| Sample-2 | 5 | ~7 |
| Sample-3 | 10 | ~8 |
| Sample-4 | 20 | ~10 |
| Sample-5 | 40 | ~17 |

*^a^*Five samples were prepared by tetragonal BaTiO_3_ nanocrystals capped with metal-free ATRP initiators as initiators (*D*: ~39 nm, Sample-5 in **Table S3**), the areal density of initiators on the surface of BaTiO_3_ nanoparticles: 2.11/nm^2^ based on TGA characterization. *^b^*The white LED irradiation time at room temperature. *^c^*Thickness of PMMA shell determined by TEM.

**References and notes:**

1. Wang, X. et al. Facile synthesis of size-tunable superparamagnetic/polymeric core/shell nanoparticles by metal-free atom transfer radical polymerization at ambient temperature. *RSC Adv.* **7**, 7789-7792 (2017).

2. Pang, X. et al. Block copolymer/ferroelectric nanoparticle nanocomposites. *Nanoscale* **5**, 8695-8702 (2013).

3. Tsuyumoto, I., Kobayashi, M., Are, T. & Yamazaki, N. Nanosized Tetragonal BaTiO_3_ Powders Synthesized by a New Peroxo-Precursor Decomposition Method. *Chem. Mater.* **22**, 3015-3020 (2010).

4. Pang, X., Zhao, L., Han, W., Xin, X. & Lin, Z. A general and robust strategy for the synthesis of nearly monodisperse colloidal nanocrystals. *Nat. Nano.* **8**, 426-431 (2013).
